# Validation of a newly proposed histopathological classification in Japanese patients with anti-neutrophil cytoplasmic antibody-associated glomerulonephritis

**DOI:** 10.1186/1471-2369-14-125

**Published:** 2013-06-17

**Authors:** Takashi Iwakiri, Shouichi Fujimoto, Kiyoki Kitagawa, Kengo Furuichi, Junya Yamahana, Yunosuke Matsuura, Atsushi Yamashita, Shigehiro Uezono, Yoshiya Shimao, Shuichi Hisanaga, Takeshi Tokura, Takashi Wada, Kazuo Kitamura, Yujiro Asada

**Affiliations:** 1 Department of Pathology, University of Miyazaki, Miyazaki, Japan; 2 Department of Hemovascular Medicine and Artificial Organs, University of Miyazaki, 5200 Kihara, Kiyotake, Miyazaki 889-1692, Japan; 3 Division of Blood Purification, Kanazawa University Hospital, Kanazawa, Japan; 4 Department of Internal Medicine, Toyama Prefectural Central Hospital, Toyama, Japan; 5 Department of Internal Medicine, Miyazaki Prefectural Hospital, Miyazaki, Japan; 6 Department of Pathology, Miyazaki Prefectural Hospital, Miyazaki, Japan; 7 Department of Internal Medicine, Koga General Hospital, Miyazaki, Japan; 8 Department of Internal Medicine, Miyazaki Social Insurance Konan Hospital, Miyazaki, Japan; 9 Division of Nephrology, Department of Laboratory Medicine, Kanazawa University, Kanazawa, Japan; 10 Division of Circulation and Body Fluid Regulation, Faculty of Medicine, University of Miyazaki, Miyazaki, Japan

**Keywords:** Anti-neutrophil cytoplasmic antibody, Histopathological classification, Immunohistochemistry, α-Smooth muscle actin

## Abstract

**Background:**

A new histopathological classification of anti-neutrophil cytoplasmic antibody (ANCA)-associated glomerulonephritis was recently proposed. We evaluated the predictive value of this classification for renal outcome in Japanese patients.

**Methods:**

We enrolled 122 patients with ANCA-associated glomerulonephritis diagnosed at several institutions in Japan between January 2000 and March 2010. Twenty patients were excluded because of observation durations of <1 year, and/or because their biopsy specimens contained <10 glomeruli. Renal biopsy specimens were categorized into four classes according to the proposed classification. We evaluated the predictive value of immunohistochemical staining for α-smooth muscle actin (SMA), Wilm’s tumor 1 (WT1), CD68, and cytokeratin for end-stage renal disease (ESRD).

**Results:**

The study population included 54 men and 48 women. Age, estimated glomerular filtration rate (eGFR), and proteinuria were 66.3 ± 11.3 years, 21.6 ml/min. and 1.10 g/24 h, respectively. Eighty-six patients were positive for myeloperoxidase-ANCA, five were positive for proteinase 3-ANCA, and 11 were negative for both antibodies. Median follow-up time was 41.0 months. Twenty-three patients (22.5%) developed ESRD during the follow-up period. Twelve patients died during follow up; 7/12 patients developed ESRD before death, and 5/12 patients died without ESRD. The incidence of ESRD increased with sequential categories: focal, 2/46 (4.3%); crescentic, 9/32 (28%); mixed, 8/18 (44%); and sclerotic, 4/6 (67%). The focal class had the best renal survival and the sclerotic class had the worst renal survival (*p* < 0.001). Kaplan-Meier renal survival analysis was similar to that of the new classification system proposal. In the multivariate analysis, the classification system tended to be a prognostic factor for ESRD (p = 0.0686, crescentic, mixed and sclerotic vs. focal, hazard ratio (HR) [95% confidence interval, CI]; 2.99 [0.61–22.7], 5.04 [1.11–36.4] and 9.93 [1.53–85.7], respectively). α-SMA-positivity also tended to be associated with ESRD (*p* = 0.1074).

**Conclusion:**

The new histopathological classification was associated with eGFR at 1 year and tended to be associated with ESRD in our Japanese cohort with ANCA-associated glomerulonephritis. α-SMA positivity might be an additional prognostic factor for ESRD.

## Background

Anti-neutrophil cytoplasmic antibody (ANCA)-associated glomerulonephritis is a common cause of rapidly progressive glomerulonephritis in adults [1]. Three major categories have been defined: microscopic polyangiitis (MPA), granulomatosis with polyangiitis (GPA), and eosinophilic granulomatosis with polyangiitis (EGPA) [2,3]. The histopathological findings are characterized by various lesions, particularly extracapillary proliferation and fibrinoid necrosis; however, lesions such as fibrous crescent formation and focal or global glomerular sclerosis, indicating chronic lesions, are also observed [4]. The renal prognosis of ANCA-associated glomerulonephritis differs greatly between individual patients. Several studies have determined the clinical and histopathological predictors of renal outcome, and shown that low levels of serum creatinine (SCr) at diagnosis and a high percentage of normal glomeruli were predictors for a better renal outcome, whereas a high percentage of sclerotic glomeruli was a predictor for a worse renal outcome [5]–[8]. However, a standardized histopathological classification of this disease has been lacking for many years.

A histopathological classification of ANCA-associated glomerulonephritis was recently proposed, based on an analysis of 100 patients from multiple centers in Europe [9]. This classification system is based on glomerular pathology and defines four classes: focal, crescentic, mixed and sclerotic. The authors reported that the phenotypic order of the class corresponded to progressive worsening of renal function. Japanese patients with ANCA-associated vasculitis show a different distribution of ANCA subtypes compared with patients from Western countries, with higher rates of myeloperoxidase (MPO)-ANCA expression than proteinase 3 (PR3)-ANCA [10,11]. We therefore performed a validation study to determine whether this proposed histopathological classification could be applied to Japanese patients with ANCA-associated glomerulonephritis.

## Methods

### Patients

We enrolled 122 patients diagnosed with biopsy-confirmed ANCA-associated glomerulonephritis between January 2000 and March 2010 from six institutions in Japan (Miyazaki University, Kanazawa University, Toyama Prefectural Central Hospital, Miyazaki Prefectural Hospital, Koga General Hospital and Miyazaki Social Insurance Konan Hospital). Patients with primary renal vasculitis were defined in accordance with the following criteria: new patients with MPA, GPA, EGPA or renal limited vasculitis (RLV) and renal involvement (elevated SCr, hematuria, proteinuria, or red cell casts) attributable to active vasculitis with or without other organ involvement, and positive serology for ANCA. Histological confirmation of renal involvement was the finding of necrotizing vasculitis and pauci-immune crescentic glomerulonephritis in the renal biopsy. The European Medicines Agency (EMEA) algorithm [12] was used to define MPA, GPA and EGPA. This algorithm utilizes the American College of Rheumatology criteria (1990) and the Chapel Hill Consensus Conference definitions. Using this approach, RLV is placed within MPA. ANCA-negative patients were eligible for enrollment in this study if there was histological confirmation of renal involvement. Patients who were followed up for <1 year (*n* = 14) or whose biopsy specimens contained <10 glomeruli (*n* = 6) were excluded. A total of 102 patients were therefore included in this study. Most of the patients received intravenous and/or oral methylprednisolone therapy with or without immunosuppressants after diagnosis, according to the attending physician’s judgement. The ethical committee of University of Miyazaki approved this study (approval number: 2010–751).

### Treatment

In this present study, 83 (81.4%) cases received methylprednisolone pulse therapy, and 89 (87.3%) cases were administered oral prednisolone. Furthermore, 30 (29.4%) cases were treated with cyclophosphamide (CYC): 15 (14.7%) were treated with intravenous CYC pulse therapy (IV-CY), 11 (10.8%) with oral CYC therapy, and four (3.9%) with both IV-CY and oral CYC. Twenty-one (20.6%) cases were treated with mizoribine (MZB), three (2.9%) cases were treated with azathioprine (AZA), and one (1.0%) case was treated with gusperimus hydrochloride.

### Patient parameters and outcomes

This was a retrospective study based on clinical information obtained from hospital records and general practice. SCr and the degree of proteinuria were measured at the time of renal biopsy, prior to the initiation of immunosuppressive therapy or hemodialysis. SCr was evaluated every 1–3 months after the initiation of therapy. The development of ESRD during follow-up was the primary outcome. ESRD was defined as requiring permanent renal replacement therapy. Estimated glomerular filtration rate (eGFR) at 1 year was the secondary outcome.

### Calculation of eGFR

SCr was assayed by an enzymatic method and eGFR was calculated using the following equation [13]: eGFR (ml/min/1.73 m^2^) = 194 × age (years)^–0.287^ × serum creatinine (mg/dl)^–1.094^ (× 0.739 for women).

### Renal histopathology

Renal biopsy specimens were examined by light microscopy and immunofluorescence analysis. For light microscopy, tissues were stained with hematoxylin and eosin, periodic acid-Schiff (PAS), AZAN, and methenamine silver. The median number of glomeruli per biopsy was 19.0 (range 10–61). For immunofluorescence analysis, sections were incubated with antibodies against IgG, IgA, IgM, C3, C4, C1q, and fibrinogen, to exclude other renal diseases. Renal biopsies were categorized into four classes in accordance with the newly proposed histopathological classification. Samples with ≥50% normal glomeruli were classified as focal, those with ≥50% cellular crescentic glomeruli were classified as crescentic, and those with ≥50% globally sclerotic glomeruli were classified as sclerotic. The other cases, those with <50% normal, cellular crescentic, and globally sclerotic glomeruli were classified as mixed. Two independent nephropathologists (T.I. and K.K.) who were blinded to the patients’ characteristics performed the histopathological evaluations and classified the specimens into the four classes. Inter-observer variation of categorization according to the new histopathological classification was tested (κ = 0.91). Discrepancies between the observers were resolved by conference to achieve consensus.

### Immunohistochemical analysis

Immunohistochemical staining was performed on samples from 34 patients with crescentic or mixed class for α-smooth muscle actin (SMA; myoepithelial marker; clone: 1A4, mouse monoclonal antibody; Dako, Glostrup, Denmark), Wilm’s tumor 1 (WT1; podocyte marker; clone: 6F-H2, mouse monoclonal antibody; Dako), CD68 (macrophage marker; clone: PG-M1, mouse monoclonal antibody; Dako) and cytokeratin (epithelial marker; clone: AE1/AE3, mouse monoclonal antibody; Dako), using 4-μm-thick sections of formalin-fixed and paraffin-embedded tissue. Sections were subsequently stained with Envision (Dako). Horseradish peroxidase activity was visualized using 3,3′-diaminobenzidine tetrahydrochloride, and counterstaining with Meyer’s hematoxylin. Of the 50 cases in either the crescentic or mixed class, 16 cases did not have sufficient tissue remaining for immunohistochemical analysis.

Immunoreactivity was evaluated by two investigators unaware of the patients’ characteristics (T.I. and A.Y.). A cell was considered positive for α-SMA, CD68 and/or cytokeratin in case of cytoplasmic staining. A cell was considered positive for WT1 in case of nuclear staining. We assessed normal glomeruli in each sample and counted all immunopositive cells. The definition of a normal glomerulus was a glomerulus without collapse, crescent formation, adhesion, or focal to segmental/global glomerulosclerosis. Even if the α-SMA-positive area was very small, the glomerulus was considered to be immunopositive. Reproducibility was assessed by blinded replicate counting of immunopositive cells performed by the two observers; the inter-observer correlation coefficients for α-SMA, WT1, CD68 and cytokeratin were r = 0.86, 0.77, 0.91, 0.87, respectively. Discrepancies between the observers were resolved by conference to achieve consensus.

### Statistical analysis

Data were analyzed using the Mann–Whitney test, Fisher’s exact test, and Kruskal-Wallis test and post hoc analysis (Dunn’s multiple comparison test) as appropriate. Kaplan-Meier survival analysis was used to compare renal survival. Multivariate analysis of renal survival was performed by Cox regression analysis. Patients who died before developing ESRD were treated as censored in Kaplan-Meier renal survival analysis and Cox regression analysis. Values of *p* < 0.05 were considered to be statistically significant. JMP 8.0.1 (SAS, Cary, NC, USA) and GraphPad Prism 5.01 (GraphPad Software, San Diego, CA, USA) were used for statistical analyses.

## Results

### Clinical and histopathological studies

**Figure 1:**
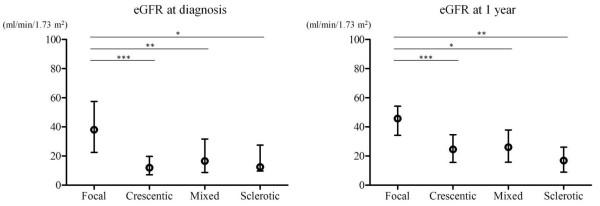
**Association between histopathological class and eGFR.** eGFRs at diagnosis and 1 year after diagnosis were higher in the focal class than in the other classes. There were no significant differences in eGFR among the crescentic, mixed, and sclerotic class. Abbreviations: eGFR, estimated glomerular filtration rate; blank circles, median values; error bars, interquartile ranges. *p < 0.05, **p < 0.01, and ***p < 0.001 (post hoc analysis).

Overall, 23 patients developed ESRD that required maintenance hemodialysis therapy during follow-up [median, 41.0 months, interquartile range (IQR), 20.0–63.8]. The baseline characteristics at the time of diagnosis for patients with and without ESRD are shown in Table 1. The mean age (± standard deviation) was 66.3 ± 11.3 years. There were 54 males and 48 females. All patients underwent serologic tests for MPO-ANCA and PR3-ANCA using enzyme-linked immunosorbent assays. Eighty-six patients were positive for MPO-ANCA and five patients were positive for PR3-ANCA; none were positive for both antibodies. Although 11 patients were negative for both antibodies, their disease was confirmed by typical histopathological features and clinical findings. The median SCr measured at the time of renal biopsy before starting immunosuppressive therapy or hemodialysis was 1.95 mg/dl (IQR, 1.30–4.15) and the median eGFR was 21.6 ml/min/1.73 m^2^ (IQR, 11.5–39.5). The median proteinuria was 1.10 g/24 h (IQR, 0.35–2.12) based on 24-h urine collection. Age, sex, serum albumin and serum C-reactive protein (CRP) were not significantly different between patients with or without ESRD. However, eGFR and 24-h proteinuria at diagnosis did differ significantly between patients with and without ESRD [eGFR: 25.7 versus (vs.) 9.0 ml/min/1.73 m^2^, *p* < 0.001; proteinuria: 1.8 vs. 0.7 g/24 h, *p* < 0.01]. According to the EMEA algorithm, 97 cases were classified as MPA, three as GPA and two as EGPA. Systemic organ involvements were as follow: ear, nose and throat, six patients (5.9%); respiratory system, 20 patients (19.6%); and nervous system, 12 patients (11.8%). The 102 patients were then categorized into four classes according to the newly proposed histopathological classification: 46 patients with focal, 32 patients with crescentic, 18 patients with mixed, and six patients with sclerotic class. The clinical and histopathological findings in the four histopathological classes are shown in Table 2. There were no significant differences in age or gender among the four classes. PR3-ANCA-positive patients occurred in the focal class (*n* = 3) and the crescentic class (*n* = 2), but not in the mixed or sclerotic class. Serum CRP was higher in the focal and crescentic class than in the mixed and sclerotic class. Proteinuria was lower in the focal class than in the other three classes. The proportion of normal glomeruli was higher in the mixed class than in the crescentic class, but the proportion of sclerotic glomeruli was also higher in the mixed class than in the crescentic class. At the time of diagnosis, the median eGFRs in the focal, crescentic, mixed, and sclerotic class were 38.1, 12.0, 16.5, and 12.4 ml/min/1.73 m^2^, respectively. eGFR was thus significantly different in the focal class compared with the other classes (*p* < 0.001 vs. crescentic; *p* < 0.01 vs. mixed; *p* < 0.05 vs. sclerotic). However, there were no significant differences among the crescentic, mixed, and sclerotic class. One year after the initial diagnosis, the median eGFRs were 45.7, 24.5, 26.0, and 16.9 ml/min/1.73 m^2^ in the focal, crescentic, mixed, and sclerotic class, respectively. Except for patients who developed ESRD, eGFR at 1 year showed an improvement from that at diagnosis in all four classes. Again, eGFR differed significantly between the focal class and the other classes (*p* < 0.001 vs. crescentic; *p* < 0.05 vs. mixed; *p* < 0.01 vs. sclerotic), but there were no significant differences among the crescentic, mixed and sclerotic class (Figure 1).

**Table 1:** Patient characteristics at the time of diagnosis

	**Total (*n*** **= 102)**	**Non-ESRD (*n*** **= 79)**	**ESRD during follow-up (*n*** **= 23)**	***p *value**
Age (years)	66.3 ± 11.3	66.3 ± 12.2	66.3 ± 7.7	n.s.
Male/female, *n*	54/48	39/40	15/8	n.s.
MPO-ANCA, *n*	86	70	16	
PR3-ANCA, *n*	5	4	1	
Both negative, *n*	11	5	6	
eGFR (ml/min/1.73 m^2^)	21.6 (11.5–39.5)	25.7 (17.4–50.4)	9.0 (5.1–13.3)	<0.001
Serum albumin (g/dl)	3.1 (2.7–3.7)	3.1 (2.7–3.7)	3.1 (2.7–3.8)	n.s.
Serum CRP (mg/dl)	3.35 (0.98–8.98)	3.70 (1.08–10.0)	2.13 (0.20–6.70)	n.s.
Proteinuria (g/day)	1.10 (0.35–2.12)	0.70 (0.30–1.77)	1.80 (1.10–2.50)	<0.01

Data are expressed as mean ± SD or median (interquartile range). *p* values were calculated using the Mann–Whitney U test or Fisher’s exact test. Statistical significance was defined as *p* < 0.05.

Abbreviations: *ESRD* end-stage renal disease, *ns* not significant, *eGFR* estimated glomerular filtration rate, *MPO* myeloperoxidase, *PR3* proteinase-3, *CRP* C-reactive protein.

**Table 2:** Baseline characteristics of patients in each histopathological class

	**Focal**	**Crescentic**	**Mixed**	**Sclerotic**	***p *value**
Age (years)	66.1 ± 11.4	66.3 ± 12.5	66.7 ± 10.2	67.5 ± 8.6	n.s.
Male/female, *n*	24/22	15/17	10/8	5/1	n.s.
MPO-ANCA, *n*	40	26	15	5	n.s.
PR3-ANCA, *n*	3	2	0	0	
Both negative, *n*	3	4	3	1	
eGFR (ml/min/1.73 m^2^)	38.1 (22.5–57.4)	12.0 (7.1–19.7)	16.5 (8.7–31.6)	12.4 (9.8–27.4)	<0.0001
Serum albumin (g/dl)	3.4 (2.8–3.7)	2.7 (2.4–3.3)	3.1 (2.8–3.7)	3.8 (3.5–3.9)	<0.01
Serum CRP (mg/dl)	4.13 (1.43–9.62)	4.98 (1.65–14.0)	1.21 (0.10–3.93)	0.16 (0.10–1.10)	<0.001
Proteinuria (g/day)	0.35 (0.21–0.79)	1.50 (1.04–2.50)	1.75 (1.07–2.20)	1.50 (0.25–3.98)	<0.0001
Immunosuppressants, yes/no, *n*	25/21	17/15	4/14	1/5	<0.05
Histopathological findings					
Normal glomeruli (%)	73.2 (60.0–81.6)	17.9 (10.6–28.0)	35.4 (22.5–43.8)	11.3 (0–22.0)	<0.0001
Crescentic glomeruli (%)	13.8 (8.9–23.6)	67.7 (60.0–74.7)	31.4 (15.6–40.4)	12.4 (3.3–22.4)	<0.0001
Globally sclerotic glomeruli (%)	6.5 (0–13.4)	3.2 (0–13.1)	29.3 (19.0–40.4)	64.5 (54.9–71.1)	<0.0001

Data are expressed as mean ± SD or median (interquartile range).

Abbreviations: *ns* not significant, *eGFR* estimated glomerular filtration rate, *MPO* myeloperoxidase, *PR3* proteinase-3, *CRP* C-reactive protein Immunosuppressants were administered after the diagnosis of ANCA-associated glomerulonephritis.

eGFR: focal vs. crescentic***, focal vs. mixed**, focal vs. sclerotic*, Serum albumin: focal vs. crescentic*, crescentic vs. sclerotic**, Serum CRP: focal vs. mixed*, focal vs. sclerotic*, crescentic vs. mixed*, crescentic vs. sclerotic**, Proteinuria: focal vs. crescentic***, focal vs. mixed**, Immunosuppressants: focal vs. mixed*, crescentic vs. mixed*, Normal glomeruli: focal vs. crescentic***, focal vs. mixed***, focal vs. sclerotic***, Sclerotic glomeruli: focal vs. crescentic***, crescentic vs. mixed***, crescentic vs. sclerotic***, Globally sclerotic glomeruli: focal vs. mixed***, focal vs. sclerotic***, crescentic vs. mixed***, crescentic vs. sclerotic***, *p < 0.05, **p < 0.01, ***p < 0.001.

**Figure 2:**
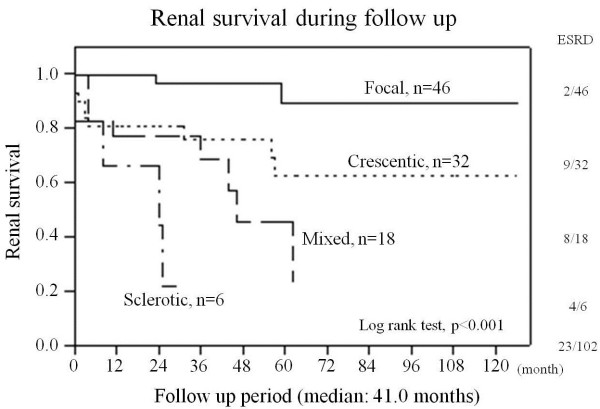
**Renal survival during follow up.** Overall, 23/102 patients developed ESRD during the total follow-up period. Renal survival was best in the focal class and worst in the sclerotic class. There was no significant difference between crescentic and mixed class regarding renal survival.

At 1 year after initial diagnosis, SCr had doubled in two patients, and another 10 patients had developed ESRD. The incidences of these events combined at 1 year were 2.2% (1/46), 21.9% (7/32), 11.1% (2/18), and 33.3% (2/6) in the focal, crescentic, mixed, and sclerotic class, respectively. During the entire follow-up period, 23 patients (22.5%) developed ESRD and 12 patients died. Seven of the 12 patients who died developed ESRD before death, and the remaining five died without ESRD. The proportions of patients who developed ESRD during follow up increased with sequential category, [focal, 4.3% (2/46); crescentic, 28.1% (9/32); mixed, 44.4% (8/18); sclerotic, 66.6% (4/6); Table 3]. Kaplan-Meier analysis of renal survival showed that prognosis was best for patients in the focal class and worst for patients in the sclerotic class (*p* < 0.001). Kaplan-Meier analysis found no significant difference between the crescentic and mixed class (*p* = 0.2716). However, the Kaplan-Meier renal survival curve was almost similar to the curve of Berden *et al.*[9] (Figure 2). In the multivariate Cox regression analysis, the new classification system at diagnosis tended to be a prognostic factor for ESRD during the follow-up period (*p* = 0.0686, crescentic, mixed and sclerotic vs. focal, hazard ratio (HR) [95% confidence interval, CI]; 2.99 [0.61–22.7], 5.04 [1.11–36.4] and 9.93 [1.53–85.7], respectively). Additionally, eGFR at diagnosis also tended to be a prognostic factor for ESRD during the follow-up period (*p* = 0.0619, HR [95% CI]; 0.97 [0.93–1.00]) (Table 4). Analysis of the incidence of ESRD according to treatment did not identify immunosuppressant use as a prognostic factor for ESRD (*p* = 0.8390, HR [95%CI]; 0.90 [0.31–2.45]). In addition, there was no significant difference between patients treated with or without CYC (data not shown).

**Table 3:** Proportions of ESRD according to the histopathological classes

**Class**	**1 year after diagnosis**	**Total follow-up period**
Focal	1/46 (2.2%)	2/46 (4.3%)
Crescentic	7/32 (21.9%)	9/32 (28.1%)
Mixed	2/18 (11.1%)	8/18 (44.4%)
Sclerotic	2/6 (33.3%)	4/6 (66.6%)

**Table 4:** Multivariate analysis of progression to ESRD

**Variable**	***p *value**	**HR (95% CI)**
Age	0.2336	1.03 (0.98–1.09)
Male	0.1072	2.33 (0.84–7.08)
eGFR at diagnosis	0.0619	0.97 (0.93–1.00)
Proteinuria at diagnosis	0.1059	1.23 (0.95–1.54)
Immunosuppressant, yes/no	0.8390	0.90 (0.31–2.45)
Histopathological classification	0.0686	crescentic vs. focal, 2.99 (0.61–22.7)
		mixed vs. focal, 5.04 (1.11–36.4)
		sclerotic vs. focal, 9.93 (1.53–85.7)

Abbreviations: *ESRD* end-stage renal disease, *HR* hazard ratio, *CI* confidence interval.

### Immunohistochemical study

**Figure 3:**
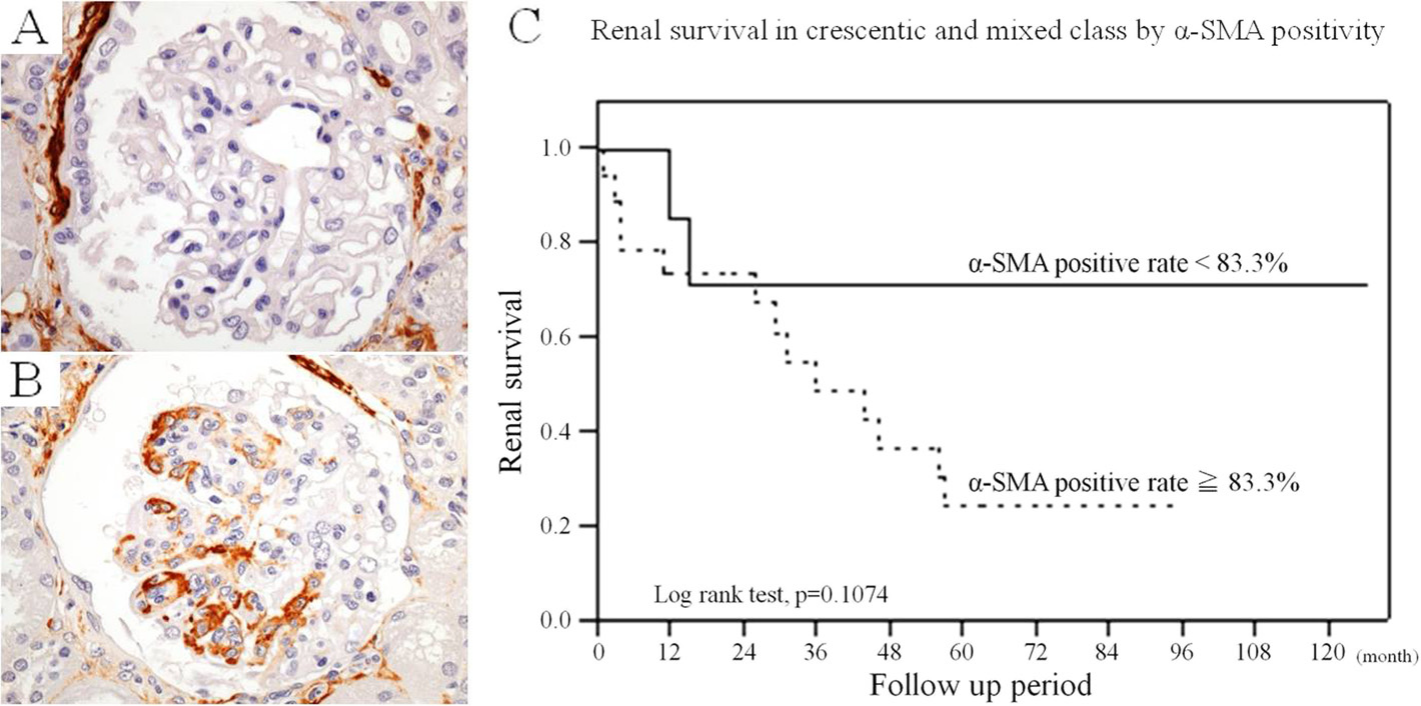
**Comparison of renal survival according to α-SMA expression in crescentic and mixed classes. (A)** Tissue section showing absence of α-SMA staining in a normal glomerulus, except in the glomerular vascular pole and thickened Bowman’s capsule. **(B)** Tissue section showing marked α-SMA expression in a normal glomerulus, suggesting activation of a mesangial cell. **(C)** Higher α-SMA positivity tended to be associated with poor renal survival. Abbreviation: SMA, smooth muscle actin.

Because histopathological evaluation by light microscopy could not differentiate the crescentic and mixed class in terms of ESRD, we evaluated the ability of immunohistochemical studies to predict renal outcome. We performed immunohistochemical staining for α-SMA, WT1, CD68, and cytokeratin on samples from 34 patients with crescentic or mixed class. There were no significant differences in expression levels of WT1, CD68, and cytokeratin between patients with and without ESRD (data not shown). By contrast, α-SMA immunoreactivity differed substantially between each normal glomerulus. As shown in Figure 3A, there was no immunoreactivity for α-SMA in this normal glomerulus, except in the glomerular vascular pole and surrounding Bowman’s capsule. On the other hand, Figure 3B shows marked immunoreactivity for α-SMA in a normal glomerulus. We counted the number of normal glomeruli in each case and then assessed if they were immunopositive for α-SMA or not. The mean proportion of α-SMA-positive glomeruli per normal glomeruli was 82.9%. Receiver operating characteristics analysis was used to identify the optimal cut-off rate of α-SMA-positive glomeruli per normal glomeruli for distinguishing renal outcome. A cut-off score of 83.3% optimally identified 100% of ESRD cases (sensitivity) and 46.7% of non-ESRD cases (specificity). In Kaplan-Meier analysis, higher α-SMA expression tended to be associated with poorer renal survival (Figure 3C).

## Discussion

### Clinical and histopathological findings

Several attempts have been made to establish histopathological criteria for ANCA-associated glomerulonephritis and to predict ESRD and overall survival in this disease [14,15]. However, studies investigating the clinical and histopathological predictors of renal outcome have provided different results with some overlap, in terms of the predictive values of normal glomeruli, glomerulosclerosis and eGFR at diagnosis [4,5,16]–[19]. Most of the proposed classification systems are cumbersome and require histopathological evaluation as well as clinical information. Berden *et al*. recently investigated the prognostic value of a simple histopathological classification, based on an analysis of 100 patients across multiple centers in Europe [9]. We examined the suitability of this classification system for Japanese patients with ANCA-associated glomerulonephritis. This histopathological classification was associated with eGFR at 1 year in our cohort, as in Berden *et al*.’s study. The incidence of ESRD during the follow-up period increased with sequential category of the proposed classification. Although the Kaplan-Meier survival curves showed the same distribution as the study by Berden *et al.*, the histopathologic classification was not associated with ESRD during follow up in our cohort in the multivariate analysis. We think there are several possible explanations for why we did not find an association between the classification and developing ESRD. First, Berden *et al*.’s study was conducted in Europe, and of the 100 patients, 45 were positive for PR3-ANCA and 47 were positive for MPO-ANCA. Notably, the distribution of ANCA subtypes in Japanese patients with ANCA-associated vasculitis differs from that in Western countries. For example, Japanese patients are more frequently positive for MPO-ANCA than PR3-ANCA [10,11]. Indeed, our cohort consisted of 86 patients with MPO-ANCA-positive disease and just five with PR3-ANCA-positive disease, while the remaining 11 patients were negative for both antibodies. The different distributions of MPO- and PR3-ANCA between Europe and Japan may thus have contributed to the conflicting results. However, we were unable to conclude if the mechanism of glomerular injury was due to the different type of ANCA, though patients with MPO-ANCA do show more active and chronic lesions than PR3-ANCA patients [20]. In addition, differences in genetic backgrounds may affect the renal pathology findings and clinical courses in patients with ANCA vasculitis. International collaborative studies are clearly needed to determine the causes of the different phenotypes and clinical courses among different populations.

A second possible reason for the apparent discrepancy between the current results and those of Berden *et al*. [9] may be the difference in treatments used in Europe and Japan. In Europe, glucocorticoids in combination with CYC or methotrexate is the standard therapeutic strategy for ANCA-associated vasculitis [21], while in Japan, although glucocorticoids in combination with CYC is also recommended as a treatment for ANCA-associated vasculitis depending on the severity of disease, CYC is only used in 33.7–45.2% of Japanese patients with ANCA-associated systemic vasculitis [22]. CYC is used less frequently in Japan to avoid associated events of infection, though its low usage may also facilitate disease remission.

Age, sex, and eGFR at diagnosis have been reported to be important predictors of renal outcome [5]–[8,18,23]. However, the current study found no significant differences in these factors at diagnosis between the crescentic and mixed class. Other reports have confirmed the importance of normal glomeruli for better renal outcome [6,8,18]. In our series, the proportion of normal glomeruli was higher in the mixed class than in the crescentic class, but the proportion of sclerotic glomeruli was also higher in the mixed class than in the crescentic class. These features probably hindered discrimination between the crescentic and mixed class.

Several studies found that immune complex or complement deposition in renal histopathology was associated with lower initial renal function, higher levels of proteinuria and poor renal outcome [24]–[26]. In the current study, only nine patients showed C3 deposition in glomerular and/or arteriolar walls. The eGFR at diagnosis, proteinuria and incidence of ESRD in these nine cases did not differ from those without C3, other immune complex or complement deposition.

### Immunohistochemical findings

There was no significant difference regarding the development of ESRD between the crescentic and mixed class. Therefore we performed immunohistochemical staining on samples from 34 patients with crescentic or mixed class to examine whether immunohistochemical factors are useful to predict ESRD in ANCA-associated glomerulonephritis. The rates of WT1, CD68, and cytokeratin expression did not differ significantly between patients with or without ESRD (data not shown). However, Kaplan-Meier analysis demonstrated that higher α-SMA immunoreactivity tended to be associated with poorer renal survival in crescentic and mixed class.

Several studies have provided evidence to suggest that glomerular component cells undergo mesenchymal transition in a pro-inflammatory environment. Epithelial-mesenchymal transition (EMT) is widely accepted as a mechanism whereby injured epithelial and/or endothelial cells transform into mesenchymal cells, which contribute to the development of fibrosis [27,28]. EMT and myofibroblastic differentiation were originally thought to occur predominantly in the interstitium; however, several reports have shown that mesangial cells can also differentiate into myofibroblasts, resulting in fibrosis [28,29]. Barnes *et al*. [30] reported that myofibroblasts were mostly responsible for interstitial accumulation and consequent structural deformations associated with fibrosis. In renal disease, glomerular mesangial cells also acquire a myofibroblastic phenotype and synthesize the same matrix proteins. α-SMA expression is considered to be a useful marker of myofibroblast differentiation in several disease settings [31]–[34]. We thought that a normal glomerulus under the light microscope might switch to a fibrogenic state if the glomerulus was positive for α-SMA. Notably, glomeruli often appeared normal under light microscopy, but α-SMA positive or negative glomeruli could be detected after careful observation. Consequently, α-SMA-positive glomeruli may progress to fibrosis, glomerulosclerosis, and renal failure. However, α-SMA positivity was not associated with renal survival. It could be the low power of the α-SMA immunohistochemical analysis (*n* = 34), since the survival analysis did not reach significance but came close to it (*p* = 0. 1074). Therefore, further studies are needed to clarify whether immunohistochemical staining of α-SMA is a useful predictor for renal prognosis.

This study had several limitations. First, the sample size was relatively small, and this may have reduced the validity of the results. Second, no standard therapy was administered to all patients, and treatments were at the discretion of the attending physicians. Third, immunohistochemical analysis was not performed in all cases and we were therefore unable to perform Cox regression multivariate analysis of the relationship between α-SMA positivity and ESRD. Fourth, the association between the histopathologic classification system and eGFR at 1 year was only based on an univariate analyses. Multivariate analysis with eGFR at 1 year as outcome was not performed. Finally, this current study was a retrospective study, thus further verification by a prospective study is desired.

## Conclusions

The histopathological classification system proposed by Berden et al. [9] is a simple method, which is associated with eGFR at 1 year and tends to be associated with the incidence of ESRD in Japanese patients with ANCA-associated glomerulonephritis in our cohort. α-SMA immunopositivity shows a trend toward an association with the renal survival rates of patients classified as crescentic or mixed class. Therefore, we suggest that immunohistochemical staining for α-SMA expression, a widely used pathological method, might be useful to estimate renal outcome. Further verification of the histopathologic classification and α-SMA staining for ANCA-associated glomerulonephritis is desired.

## Competing interests

The authors declare that they have no competing interests.

## Authors’ contributions

TI, SF and KK made substantial contributions to conception and design. TI, SF, KK, JY, SU, YS, SH and TT were involved in acquisition and interpretation of data. TI, SF, YM and AY performed the statistical analysis and interpretation of data. TI, KK and AY performed histopathological evaluation. SF, KF, TW, KK and AY advised throughout the study and its final approval and helped to draft the manuscript. All authors read and approved the final manuscript.

## Pre-publication history

The pre-publication history for this paper can be accessed here:

http://www.biomedcentral.com/1471-2369/14/125/prepub
